# Significant discrepancies exist between clinician assessment and patient self-assessment of functional capacity by validated scoring tools during preoperative evaluation

**DOI:** 10.1186/s13741-016-0041-4

**Published:** 2016-07-13

**Authors:** John Whittemore Stokes, Jonathan Porter Wanderer, Matthew David McEvoy

**Affiliations:** Vanderbilt University School of Medicine, 2215 Garland Avenue (Light Hall), Nashville, TN 37232 USA; Multispecialty Adult Anesthesiology, Vanderbilt University Medical Center, 1301 Medical Center Drive, 4648 The Vanderbilt Clinic, Nashville, TN 37232-5614 USA

**Keywords:** Functional capacity, Self-triage, Preoperative assessment, Perioperative risk, Electronic questionnaire

## Abstract

**Background:**

Preoperative assessment of functional capacity is necessary to direct decisions regarding cardiac evaluation and may help identify patients at high risk for perioperative complications. Patient self-triage regarding functional capacity could be useful for discerning which patients benefit from a clinician evaluation at a Preoperative Evaluation Center prior to the day of surgery. We evaluated the feasibility of preoperative, patient self-triage regarding functional capacity.

**Methods:**

Patients were recruited immediately prior to their preoperative evaluation. Study participants completed electronic versions of the Duke Activity Status Index (DASI) and the Patient-Reported Outcomes Measurement System (PROMIS)–Short Form 12a–Physical Function. DASI and PROMIS questionnaire responses were scored and evaluated for correlation with clinician assessments of functional capacity. Correlation was analyzed around the dichotomous outcome of <4 metabolic equivalents of task (METs) or ≥4 METs. Patients also evaluated the usability of the questionnaires.

**Results:**

After IRB approval, 204 patients were enrolled and completed both DASI and PROMIS questionnaires. Clinicians assessed functional capacity at <4 METs for 109 patients (53.4 %) compared to 18 (8.8 %) patient self-assessments <4 METs as estimated by DASI. These results represent a significant discrepancy between assessments (Fisher’s exact, two-tailed *P* value <0.0001). The standard T-score of PROMIS estimates of functional capacity correlated with DASI estimates (*R*^2^ 0.76). The mean and standard deviation for PROMIS T-scores were 43.3 and 9.86, respectively (mean 50.0; SD 10.0 for the general population).

Of the 203 patients who completed the entire study survey, 192 (94.6 %) stated that they did not require assistance from another person, and 187 (94 %) responded either “agree” or “strongly agree” to the DASI questionnaire being “easy to understand” and “easy to complete;” 186 (93 %) and 188 (94 %), respectively, responded similarly to the PROMIS questionnaire.

**Conclusions:**

While both electronic questionnaires were easy to understand and complete for most study participants, there was a significant discrepancy between clinician assessments and patient self-assessments of functional capacity. Further study is needed to determine if either patient self-triage by means of activity questionnaires or clinician evaluation is valid and reliable in the preoperative setting.

**Electronic supplementary material:**

The online version of this article (doi:10.1186/s13741-016-0041-4) contains supplementary material, which is available to authorized users.

## Background

Valid and reliable assessment of functional capacity is an important component of the preoperative evaluation. Patient functional capacity directs decisions about preoperative cardiac evaluation and is useful for risk stratification prior to surgery (Fleisher et al. 2014). Poor performance on formal exercise tolerance testing reliably correlates to increased risk for perioperative complications in several different patient populations and treatment settings (Snowden et al. 2010; Wilson et al. 2010). However, because of expense and practical considerations, exercise tolerance testing is not routinely performed prior to non-cardiac surgery in the USA. Functional capacity is commonly assessed through obtaining the patient’s history regarding their ability to perform certain physical activities. Clinician-elicited stair-climbing ability has been shown to correlate to perioperative cardiac events and other complications (Reilly et al. 1999), and categorical metabolic equivalents of task (METs) estimates, as determined through clinician history of physical capabilities, have been shown in a univariate analysis to be predictive of perioperative cardiac outcomes (Wiklund et al. 2001).

Activity questionnaires, such as the Duke Activity Status Index (DASI) (Hlatky et al. 1989) and the Patient-Reported Outcomes Measurement System (PROMIS)–Short Form 12a–Physical Function (www.nihpromis.org), are available to guide clinicians when estimating METs in the preoperative assessment of functional capacity (Fleisher et al. 2014). Patient-completed versions of the DASI questionnaire have been shown to correlate moderately well with physiologic measures of functional capacity or exercise tolerance in several clinical settings (Dunagan et al. 2013; Shaw et al. 2006; Struthers et al. 2008), and patient reported exercise capacity has been shown to be predictive of survival in vascular surgery patients (Boult et al. 2015). Patient self-assessment of functional capacity by means of electronic questionnaires would allow METs estimates to be known prior to in-person, preoperative evaluations, enabling preoperative triage of patients based on estimated functional capacity, a core component of preoperative evaluation (Fleisher et al. 2014).

In this study, we sought to evaluate the feasibility patient self-triage regarding functional capacity by investigating the correlation between clinician assessments and patient self-assessment of functional capacity, as assisted by electronic, patient-completed DASI and PROMIS questionnaires. In addition, we analyzed patient survey data regarding the usability of these two validated activity questionnaires.

## Methods

### Population and enrollment

The study was approved by the Vanderbilt University Institutional Review Board. All patients, age 18 years or older, who were scheduled for elective surgery at our institution and seen in the Preoperative Evaluation Center (PEC) prior to their surgery were eligible for enrollment. Patients undergoing moderate to high-risk surgeries or who have moderate to high-risk comorbidities are referred to our PEC by their surgeon. A member of the study team recruited patients immediately prior to the preoperative evaluation and obtained written informed consent. Baseline data was not available for power analysis prior to initiating enrollment; therefore, a convenience sample of patients was recruited during the month of March 2015.

### Study questionnaire

Prior to initiation of the clinician encounter, participants were asked to independently complete electronic questionnaires on a tablet computer (iPad, Apple Inc.; Cupertino, CA). The DASI and the PROMIS questionnaires, in addition to questions to assess the comparative usability of these two formal activity questionnaires (see Additional files 1, 2, and 3), were administered using the research electronic data capture system (Harris et al. 2009). The study administrator was not present with the patients as they completed the questionnaire. Patients were asked to complete the questionnaire without assistance but were permitted help from an accompanying family member, friend, or care provider if necessary.

### Clinician evaluation and functional capacity assessment

Following completion of the study questionnaire, each patient underwent preoperative clinical evaluation. The clinicians performed and documented the evaluation in accordance with the standard practice for all preoperative consultations in the PEC at our institution. Routine documentation of PEC evaluations includes estimating functional capacity in our electronic medical record as one of five categories: excellent (>7 METs); very good (5–7 METs); good (4 METs); fair (2–3 METs); and poor (1–2 METs). As a reference tool, clinicians are provided with a list of physical activities and the METs associated with those activities as described in the 2007 American College of Cardiology/American Heart Association Guidelines on Perioperative Cardiac Evaluation and Care for Noncardiac Surgery (Fleisher et al. 2007). Additionally, clinicians have structured documentation for reasons for physical limitations, including angina, dyspnea, claudication, and fatigue, as well as the ability to provide free text descriptions of other reasons for physical function limitations. The clinicians performing the preoperative assessments were blinded to the patient responses on the DASI and PROMIS forms.

### Questionnaire scoring and data elements

At the conclusion of patient enrollment, the DASI and PROMIS questionnaire elements were scored. The DASI questionnaire was scored according to the published methodology to estimate functional capacity in terms of METs (Hlatky et al. 1989). Individual DASI questions carry different weight, and the questionnaire can be scored to produce a METs estimate from 2.74 to 9.89 METs. A raw score for the PROMIS questionnaire is generated from the responses to the five-point Likert options. Raw scores range from 6 to 60 and correlated to a standard T-score. The correlation of raw scores to standard T-scores is developed from population statistics of functional capacity.

Responses to the questions regarding the clarity and usability of the DASI and PROMIS electronic questionnaires were directly analyzed for comparative usability of these two formal activity questionnaires.

From the electronic medical record, we retrieved documented clinician estimates of functional capacity, as well as American Society of Anesthesiologists (ASA) physical status. The previously described categorical estimates of functional capacity were then compared to the results from patient-completed DASI and PROMIS electronic questionnaires. We also searched the medical record for study participants who had completed exercise tolerance testing or exercise stress testing.

### Statistical analysis

To determine the correlation between clinician assessments and patient self-assessments of functional capacity, DASI METs estimates were compared to clinician categorical assessments of functional capacity using the dichotomous categories of ≥4 METs and <4 METs. Statistical correlation was analyzed using a two-tailed, Fisher’s exact test. This METs threshold was chosen, as it is a branch point in the algorithm for preoperative evaluation of coronary artery disease, as described by the 2014 *American College of Cardiology/American Heart Association Guideline on Perioperative Cardiovascular Evaluation and Management of Patients Undergoing Noncardiac Surgery* (Fleisher et al. 2014). The T-scores from the PROMIS questionnaire results were compared to the DASI METs estimates using linear regression analysis.

## Results

After IRB approval, 211 patients consented for participation; 204 patients were eligible for inclusion in the final analysis of functional capacity assessments. Reasons for exclusion from the final analysis include failure to complete the survey (six patients) and absence of a documented clinician estimate of functional capacity (one patient). Of the six patients who did not complete the survey, two were due to clinician interruption, two were due to participant refusal to answer specific survey questions, one was due to inability to understand the questions, and one was due to accidental closure of the electronic survey application. One patient completed both the DASI and PROMIS components of the survey and then accidentally closed the electronic survey application prior to completion of the final usability field; thus, only 203 participants are included in the final usability analysis.

### Demographics

Of the 204 patients included in the final analysis of functional capacity assessments, the mean age was 56.8 (standard deviation 15.3); 32.2 % of the participants were classified as ASA I/II, 67.8 % ASA III/IV (Table 1).

**Table 1:** Patient characteristics

Age	(Mean ± SD)
	56.8 ± 15.3
Gender	*N* (%)
Male	85 (41.7)
Female	119 (58.3)
ASA classification	*N* (%)
ASA 1	1 (0.5)
ASA 2	63 (31.7)
ASA 3	129 (64.8)
ASA 4	6 (3.0)

This table describes the demographics of the study population by age, gender, and American Society of Anesthesiologists (ASA) classification

### Functional capacity estimates

**Fig. 1:**
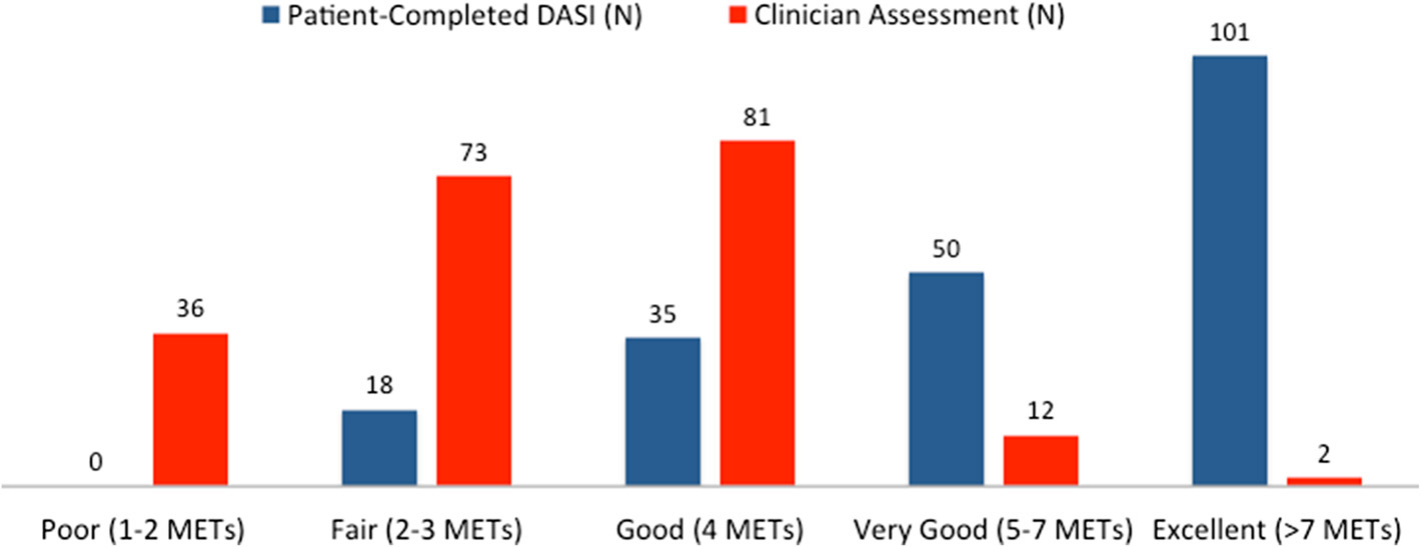
Distribution of patient and clinician METs assessment results across the study population. This figure illustrates the distribution of patient and clinician categorical metabolic equivalents of task (METs) assessment results across the study population. Here, patient METs self-assessment results were determined from their scored responses to the Duke Activity Status Index (DASI), which generates a numerical METs calculation. The categorical distribution of the DASI results is shown in the *blue columns*. Clinician METs assessments were carried out and documented in accordance with the standard practice at our Preoperative Evaluation Center (PEC). The distribution of clinician categorical METs assessments for the study population is displayed in the *red columns*

**Fig. 2:**
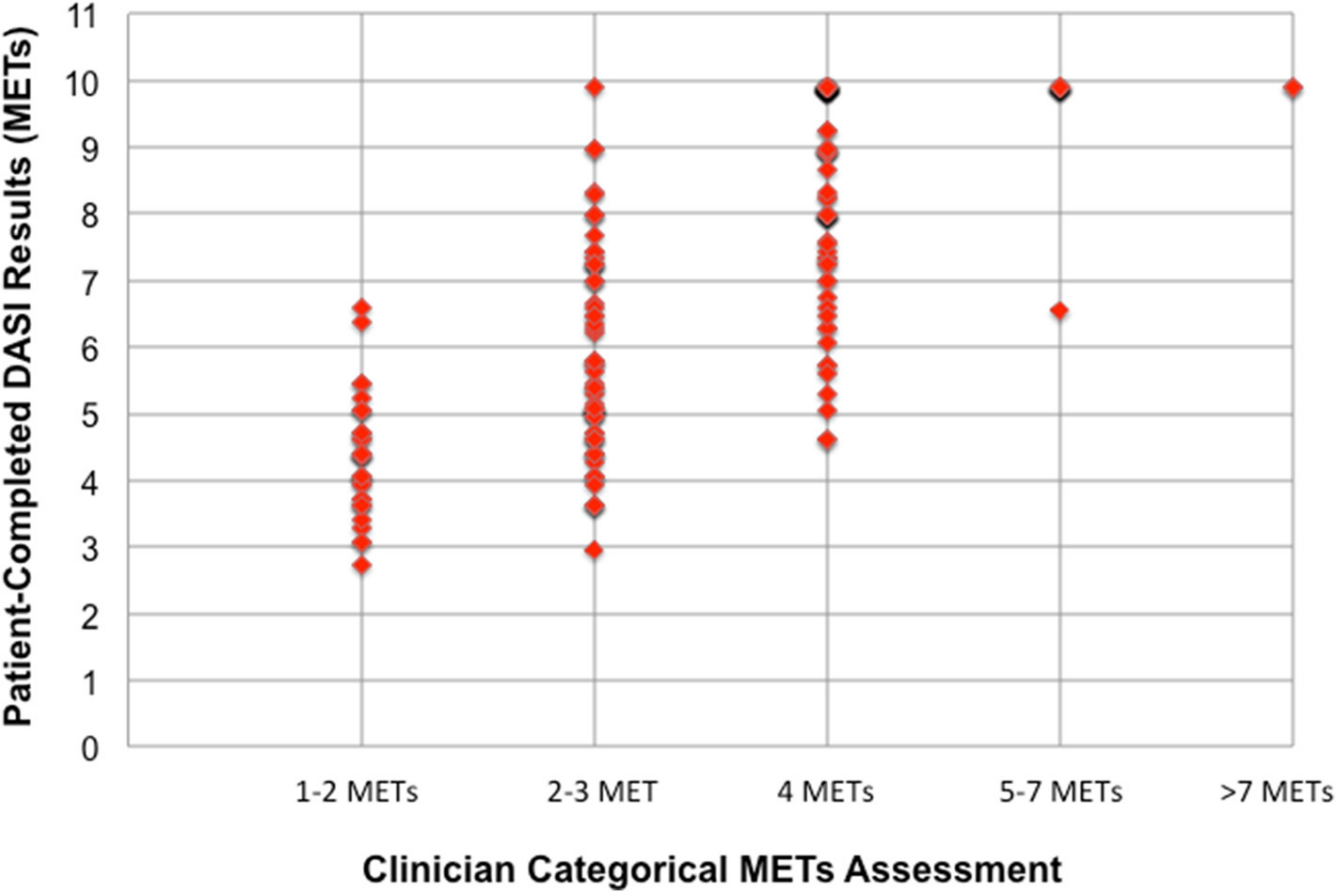
Clinician vs. patient self-assessment (DASI results). This graph displays the discrepancy between clinician categorical assessments of functional capacity and patient self-assessments of functional capacity. Once again, patient self-assessments of functional capacity were determined from their scored responses to the Duke Activity Status Index (DASI), which generates calculated functional capacity in terms of metabolic equivalents of task (METs). At our Preoperative Evaluation Center, clinician functional capacity assessments are routinely documented in terms of the categorical groupings displayed on the x-axis. In this figure, the DASI patient self-assessments are plotted against clinician assessments of functional capacity. A lack of correlation is evident in this figure, particularly around the clinically significant value of four METs of physical work

**Fig. 3:**
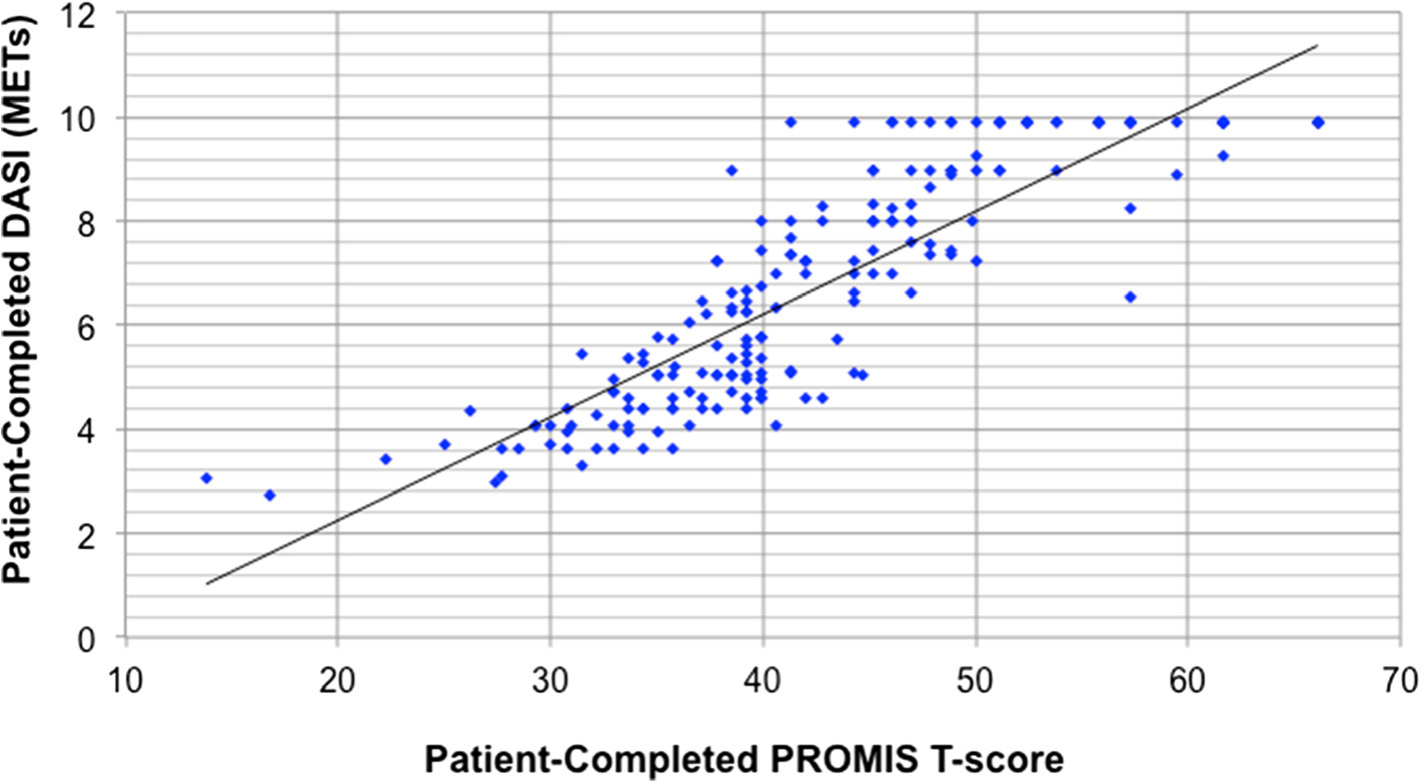
PROMIS T-score vs. DASI METs. In this figure, the T-scores of the Patient-Reported Outcomes Measurement System (PROMIS)–Short Form 12a–Physical Function results are plotted against the Duke Activity Status Index (DASI) results, as reported in terms of the calculated metabolic equivalents of task (METs). Patients completed both the PROMIS and DASI activity questionnaires prior to their clinician evaluation. For our study population, results from both of these questionnaires employed in patient self-assessments of functional capacity correlated linearly with each other (*R* ^2^ 0.76)

Clinicians assessed functional capacity at <4 METs for 109 patients (53.4 %), while only 18 patients (8.8 %) assessed their functional capacity at <4 METs, as calculated by their responses on the DASI. These results represent a significant discrepancy between assessments around the clinically relevant point of 4 METs (Fisher’s exact, two-tailed *P* value <0.0001). Graphical relationship between categorical clinician functional capacity assessments and DASI patient self-assessments is displayed in Figs. 1 and 2. The standard T-score of PROMIS estimates of functional capacity correlated linearly (*R*^2^ 0.76) with DASI estimates of functional capacity (see Fig. 3). The mean and standard deviation for PROMIS T-scores were 43.3 and 9.86, respectively (mean 50.0; SD 10.0 for the general population). No patients enrolled in our study had documentation of exercise testing; thus, comparison of clinician and patient assessments of functional capacity to physiologic measures of functional capacity was not possible.

### Usability

Of the 203 included in the usability analysis, 192 (94.6 %) stated they did not require assistance from another person. One hundred eighty-seven (94 %) responded either “agree” or “strongly agree” to the DASI questionnaire being “easy to understand” and “easy to complete,” and 186 (93 %) and 188 (94 %), respectively, responded similarly for the PROMIS questionnaire. See Table 2 for further usability data.

**Table 2:** Electronic activity questionnaire usability results

	DASI	PROMIS
	*n* (%)	*n* (%)
Independent completion	192 (94.6)	192 (94.6)
Easy to understand	187 (93.5)	186 (93.0)
Easy to complete	187 (93.5)	188 (94.0)
Which was easier to complete?	139 (68.1)	64 (31.5)
	Mean ± SD	Mean ± SD
Completion time (min:s)	01:55 ± 02:08	02:29 ± 1:42

This table displays the results of the responses to survey questions regarding the usability of the two activity questionnaires employed for patient self-triage of functional capacity, the Duke Activity Status Index (DASI), and the Patient-Reported Outcomes Measurement System (PROMIS)–Short Form 12a–Physical Function. The majority of study participants found these questionnaires easy to understand and easy to complete using a first generation iPad. The average time to complete both surveys was less than 3 min

*SD* standard deviation

## Discussion

The assessment of functional capacity is an integral component of the preoperative evaluation. We sought to determine whether patient self-assessment of functional capacity using electronic activity questionnaires is feasible and valid in the setting of preoperative evaluations. In our patient population, while both electronic questionnaires were easy to understand and complete for most study participants, there was a significant discrepancy between clinician assessments and patient assessments from formal valid questionnaires, particularly around the dichotomous result of whether or not a patient can achieve four or more METs of physical work.

### METs assessment discrepancy

The significant discrepancy between clinician and patient assessments of functional capacity in our patient population highlights that a clinician-elicited history regarding physical capabilities may be very different than physical capabilities that are purely patient-reported. This distinction was evident even in the initial validation of the DASI (Hlatky et al. 1989). The DASI was developed in two phases, a development phase in which an interviewer asked the subjects questions regarding physical function, and a validation phase in which the subjects independently completed the initial version of the DASI. In both phases, subjects underwent exercise tolerance testing after either the interview or completion of the DASI questionnaire. Both phases showed statistically significant correlation of “patient-reported” functional capacity to physiologic measures of functional capacity; however, the correlation was better in the development phase (Spearman’s correlation 0.81) than in the validation phase (Spearman’s correlation 0.58) (Hlatky et al. 1989). It may be that the personal interaction between interviewer and interviewee increases the accuracy of patient-reported physical capabilities. McGlade et al. (2001) demonstrated that patient-completed DASI scores correlated with a next of kin’s DASI assessment of functional capacity; however, patients slightly overestimated their capabilities as compared to their next of kin (McGlade et al. 2001). In our study, participants were often accompanied by a spouse, friend, child, or other care provider, who may have added to the interaction through verbal or non-verbal communication with the clinician, which could increase the accuracy of clinician functional capacity assessments.

### Reliability of patient-reported health information

Several studies have evaluated the ability of patients to identify clinical risk factors in the setting of chronic diseases and demonstrated that patient-completed surveys regarding diagnosed medical conditions are probably at least as reliable as the medical record (Tisnado et al. 2006; Okura et al. 2004). It has also been shown that Revised Cardiac Risk Index (Lee et al. 1999) scores calculated from patient survey data correlate well with scores calculated from clinician documentation of risk factors (Manaktala et al. 2013). However, these studies did not evaluate the reliability of patient-reported functional capacity, which may be different than other types of patient-reported health information.

Patients may have an increased tendency to over-estimate their physical capabilities when completing an activity questionnaire. Dunagan et al. (2013) showed slight over-estimation of METs level by the DASI questionnaire when compared to exercise tolerance testing in the setting of cardiac rehabilitation, although this over-estimation did not represent a significant difference in their sample (Dunagan et al. 2013). Formal activity questionnaires are traditionally validated by having participants complete the questionnaire and then subsequently asking patients to undergo a physiologic measure of functional capacity, such as exercise tolerance testing (Hlatky et al. 1989; Struthers et al. 2008). In the absence of being required to demonstrate physical capabilities, as was the case in our study, participants may have an increased tendency to exaggerate physical capabilities. McGlade et al. (2001) found that of 68 patients who answered affirmatively to DASI question number four regarding stair climbing, 13 patients were unable to demonstrate the ability to climb a flight of stairs (McGlade et al. 2001). The authors concluded that rather than asking patients if they are capable of a physical task, it may be more useful to ask them to demonstrate their ability to do so (McGlade et al. 2001).

It is also possible that clinician underestimation of functional capacity contributed to the discrepancy demonstrated in our study. Anecdotally, the authors have observed that clinicians often use one or two history questions in the assessment of a patient’s functional capacity. Thus, a negative response to a single question may lead a clinician to underestimate a patient’s functional capacity when a patient is capable of achieving four METs of physical work during activities not addressed by the clinician.

Additionally, while the clinician functional capacity tool employed at our PEC includes phrases to describe physical activity that are very similar or equivalent to the language used in DASI questions, the categorical METs assessments assigned to a particular group of activities does not necessarily correspond to the DASI scoring formula. Thus, it is possible for clinician and patient-completed DASI METs estimates to be discrepant, even if patients described their physical capabilities to clinicians in complete concordance with how they responded to DASI questions.

### Usability

While there is poor correlation between clinician and questionnaire estimates of functional capacity, the majority of our study participants found the electronic questionnaires easy to understand and easy to complete. Patients were able to complete these surveys on a touch-screen, tablet computer independently and in a timely manner. Our patient-entered responses to the usability of the electronic questionnaires suggest that this modality would be acceptable for application in patient-self triage if self-assessment of functional capacity using a formal activity questionnaire was validated as an accurate method of determining functional capacity in the preoperative setting.

### Limitations

As there were no physiologic measures of functional capacity obtained on patients enrolled in our study, we cannot comment on the validity of either clinician assessments or patient assessments of functional capacity relative to performance on physiologic measures of functional capacity, such as cardiopulmonary exercise testing. Without physiologic measures of functional capacity, we cannot determine whether the discrepancy between clinician and questionnaire estimates of functional capacity represents patient over-estimation or clinician underestimation of functional capacity. Similarly, without data regarding the surgical outcomes of the study participants, we cannot determine whether clinician or patient assessments of functional capacity are more clinically relevant for predicting surgical complications or major adverse cardiac events, nor can we determine the relative clinical significance patient responses to particular survey questions, such as stair-climbing ability. Whether or not patients can accurately report their functional capacity or clinicians accurately assess functional capacity, both assessments may still be useful in screening for high-risk patients (Reilly et al. 1999; Wiklund et al. 2001; Boult et al. 2015).

### Directions for future study

Electronic versions of formal activity questionnaires have theoretical potential to enable patient self-triage regarding functional capacity. As it is unclear which patients may benefit from a clinician evaluation at a PEC prior to the day of surgery, implementation of patient self-assessments using such tools could improve the efficiency and quality of preoperative evaluations while reducing costs by limiting unnecessary evaluations and testing in patients who have good functional capacity. However, further work is needed to determine whether patients accurately assess their own functional capacity in the preoperative setting using electronic versions of formal activity questionnaires. Future study is also needed to determine whether self-assessment of functional capacity by means of electronic questionnaires can predict perioperative complications or major adverse cardiac events. Finally, since functional capacity is commonly assessed by clinicians, more study is needed to determine whether clinicians accurately and reliably assess a patient’s functional capacity through routine history questions.

## Conclusions

Our patient population found electronic versions of both the DASI and PROMIS activity questionnaires easy to understand and complete, suggesting a potential for application of similar tools for patient self-triage prior to preoperative evaluations. However, we found a significant discrepancy between clinician and patient self-assessment of functional capacity. Before preoperative, patient self-triage regarding functional capacity is implemented, more study is needed to determine whether patients accurately assess their own functional capacity using activity questionnaires. Additionally, our results highlight the importance of the distinction between clinician-elicited and patient-reported functional capacity, as the two may not be equivalent.

## Abbreviations

ASA, American Society of Anesthesiologists; DASI, Duke Activity Status Index; METs, metabolic equivalents of task; PEC, Preoperative Evaluation Center; PROMIS, Patient-Reported Outcomes Measurement Information System

## Additional files

Additional file 1: Usability Survey. This file represents the survey completed by participants to assess the usability of the two formal activity questionnaires employed in this study for patient self-triage regarding functional capacity. (DOCX 73 kb) Additional file 2: Patient-Reported Outcomes Measurement System (PROMIS)–Short Form 12a–Physical Function. This file is a text version of the PROMIS–Short Form 12a–Physical Function formal activity questionnaire. (PDF 46 kb) Additional file 3: Duke Activity Status Index (DASI). This file is a text version of the Duke Activity Status Index (DASI) formal activity questionnaire. (PDF 43 kb)
