# Supplementary figures and images for: Significant discrepancies exist between clinician assessment and patient self-assessment of functional capacity by validated scoring tools during preoperative evaluation
Source: Perioper Med (Lond). 2016 Jul 13;5:18. doi: 10.1186/s13741-016-0041-4 (PMC4942938; doi:10.1186/s13741-016-0041-4)

Usability Survey


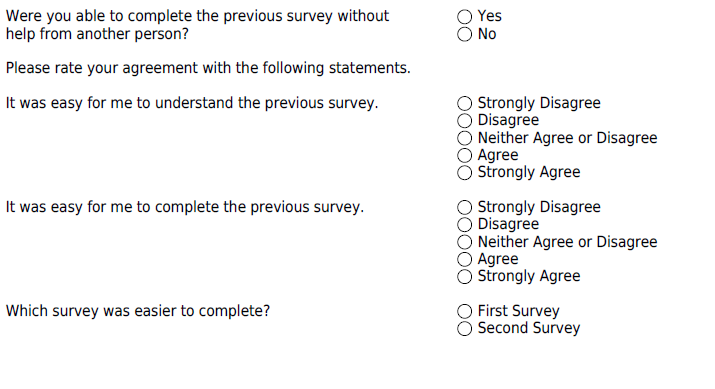

Supplement: Additional file 1: — Usability Survey. This file represents the survey completed by participants to assess the usability of the two formal activity questionnaires employed in this study for patient self-triage regarding functional capacity. (DOCX 73 kb) [file 13741_2016_41_MOESM1_ESM.docx]
