# Supplementary material for: Significant discrepancies exist between clinician assessment and patient self-assessment of functional capacity by validated scoring tools during preoperative evaluation
Source: Perioper Med (Lond). 2016 Jul 13;5:18. doi: 10.1186/s13741-016-0041-4 (PMC4942938; doi:10.1186/s13741-016-0041-4)
Supplement: Additional file 2: — Patient-Reported Outcomes Measurement System (PROMIS)–Short Form 12a–Physical Function. This file is a text version of the PROMIS–Short Form 12a–Physical Function formal activity questionnaire. (PDF 46 kb) [file 13741_2016_41_MOESM2_ESM.pdf]

Please respond to each item by choosing one answer per statement. The following questions ask about your ability to stand and move with and without support. "Support" means using items such as canes, walking sticks, walkers and leg braces, or other people.

Can you walk 25 feet on a level surface (with or without support)? ◯ Yes ◯ No

	Without any difficulty	With a little difficulty	With some difficulty	With much difficulty	Unable to do
Are you able to walk a block on flat ground?	◯	◯	◯	◯	◯
Are you able to walk up and down two steps?	◯	◯	◯	◯	◯
Are you able to run at a fast pace for two miles?	◯	◯	◯	◯	◯
Are you able to do yard work like raking leaves, weeding, or pushing a lawn mower?	◯	◯	◯	◯	◯

	Not at all	Very little	Somewhat	Quite a lot	Cannot do
Does your health now limit you in doing strenuous activities such as backpacking, skiing, playing tennis, bicycling or jogging?....	◯	◯	◯	◯	◯
Does your health now limit you in hiking a couple of miles on uneven surfaces, including hills?	◯	◯	◯	◯	◯

	Without any difficulty	With a little difficulty	With some difficulty	With much difficulty	Unable to do
Are you able to wash and dry your body?	◯	◯	◯	◯	◯
Are you able to get in and out of bed?	◯	◯	◯	◯	◯
Are you able to bend down and pick up clothing from the floor?	◯	◯	◯	◯	◯
Are you able to push open a heavy door?	◯	◯	◯	◯	◯
Are you able to reach and get down an object (such as a can of soup) from above your head?	◯	◯	◯	◯	◯

	Not at all	Very little	Somewhat	Quite a lot	Cannot do
Does your health now limit you in doing eight hours of physical labor?	◯	◯	◯	◯	◯
