# Supplementary material for: Significant discrepancies exist between clinician assessment and patient self-assessment of functional capacity by validated scoring tools during preoperative evaluation
Source: Perioper Med (Lond). 2016 Jul 13;5:18. doi: 10.1186/s13741-016-0041-4 (PMC4942938; doi:10.1186/s13741-016-0041-4)
Supplement: Additional file 3: — Duke Activity Status Index (DASI). This file is a text version of the Duke Activity Status Index (DASI) formal activity questionnaire. (PDF 43 kb) [file 13741_2016_41_MOESM3_ESM.pdf]

Survey D

	Yes	No
Are you able to take care of yourself, that is, eating, dressing, bathing, or using the toilet?	○	○
Are you able to walk indoors, such as around the house?	○	○
Are you able to walk a block or 2 on level ground?	○	○
Are you able to climb a flight of stairs or walk up a hill without stopping?	○	○
Are you able to run a short distance?	○	○
Are you able to do light work around the house like dusting or washing dishes?	○	○
Are you able to do moderate work around the house like vacuuming, sweeping floors, or carrying in the groceries?	○	○
Are you able to do heavy work around the house like scrubbing floors, or lifting or moving heavy furniture?	○	○
Are you able to do yard work like raking leaves, weeding or pushing a power mower?	○	○
Are you able to have sexual relations?	○	○
Are you able to participate in moderate recreational activities like golf, bowling, dancing, doubles tennis, or throwing a baseball or football?	○	○
Are you able to participate in strenuous sports like swimming, singles tennis, football, basketball or skiing?	○	○
